# Clinical response of pigment epithelial detachment associated with neovascular age-related macular degeneration in switching treatment from Ranibizumab to Aflibercept

**DOI:** 10.1186/s12886-018-0824-0

**Published:** 2018-06-22

**Authors:** Pallavi Tyagi, Zain Juma, Yong Keen Hor, Neil W. Scott, Andreea Ionean, Cynthia Santiago

**Affiliations:** 1 Department of Ophthalmology, Aberdeen Royal infirmary, Forresterhill Road, Aberdeen, AB25 5ZN UK; 2 The school of medicine, University of Aberdeen, Aberdeen, AB25 2ZD UK; 3 Medical Statistics Team, University of Aberdeen, Aberdeen, AB25 2ZD UK

**Keywords:** Aflibercept, Pigment epithelial detachment, Neovascular age related macular degeneration

## Abstract

**Background:**

To study the clinical outcomes of pigment epithelial detachment (PED) associated with neovascular age-related macular degeneration (nAMD) in patients switched from Ranibizumab to Aflibercept**.**

**Methods:**

Retrospective non-comparative case series. 50 eyes with active nAMD and fovea involving PED of ≥100 μm measured manually using the caliper on the OCT, initially treated with intravitreal Ranibizumab (0.5 mg/0.05 ml) and later switched to Aflibercept (2.0 mg/0.05 ml). The outcome measures of best corrected visual acuity (BCVA), PED height, PED width and number of injections were measured at baseline and at time point of switch, 4 months, 1 year and at last follow up visit post-switch. Three paired t-tests and Pearson’s correlations were calculated to analyze variables at switch and change in variables at 1 year.

**Results:**

After switch to Aflibercept, the improvement of BCVA was 1.84 (*p* = 0.11), 1.74 (*p* = 0.21) and 1.16 (*p* = 0.45) letters, the change in PED height was − 65.6μm (*p* < 0.001), − 50.64μm (*p* = 0.007) and − 68.48μm (p < 0.001) and the change in PED width was − 36.6μm (*p* = 0.514), + 29.7μm (*p* = 0.922) and + 118.4μm (*p* = 0.210) at 4 months, 1 year and the last visit respectively. There was a moderate negative correlation between reduction in PED height at 1 year after switch and PED height at the time of switch to Aflibercept (*r* = − 0.474, *p* < 0.05).

**Conclusion:**

The improvement in BCVA and change in PED width was not statistically significant however the reduction in PED height was significant after switching treatment to Aflibercept. The change in BCVA at 1 year after switch was not correlated with any of the analyzed anatomical characteristics of PED.

## Background

Clinically active neovascular age related macular degeneration (nAMD) can manifest with sub-retinal fluid (SRF), intra-retinal fluid (IRF) and retinal pigment epithelial detachment (PED). PED can be categorised clinically and angiographically into drusenoid, serous and fibrovascular [1]. Optical coherence tomography (OCT) enables qualitative and quantitative assessment of the dimensions, reflectivity, progression and response to treatment of PED lesions. The resolution of PED has largely been unsatisfactory with treatments including laser, photodynamic therapy, intraocular gas, intravitreal triamcinolone [2–4]. Currently intravitreal anti-vascular endothelial growth factor (anti-VEGF) agents are mainstay treatment for nAMD. The current focus is to assess functional and anatomical response of anti-VEGF agents as Bevacizumab (Avastin®), Ranibizumab (Lucentis®) and Aflibercept (Eylea®) on PED and also switching treatment between these agents. In the United Kingdom, Ranibizumab and Aflibercept are both licensed for treatment of nAMD [5, 6]. Ranibizumab is a humanized monoclonal antibody antigen-binding fragment, which inhibits all biologically active isoforms of VEGF-A whereas Aflibercept is a recombinant fusion protein of components of VEGF receptor and antagonizes VEGF-A, VEGF-B and placental growth factor [7]. Land mark VIEW 1 and VIEW2 studies have demonstrated equal functional and anatomical efficacy of the 2 drugs [8]. Aflibercept has been shown to have pharmacological advantage of higher binding affinity to VEGF-A and a longer ligand binding activity [9]. This theoretical advantage and later introduction of Aflibercept have led to several studies investigating the switching from ranibizumab and/or bevacizumab to aflibercept in cases of refractory or rapidly recurring fluid in nAMD. However, there is scarcity of evidence for anatomical and functional outcomes in PED following anti-VEGF therapy. The purpose of this study is to evaluate the clinical response in patients with PED associated with nAMD whose treatment was switched from Ranibizumab to Aflibercept.

## Methods

This is a retrospective non-comparative case series that was performed in Aberdeen Royal Infirmary, Aberdeen, United Kingdom.

### Inclusion and exclusion criteria

In this series we identified eyes with active nAMD and PED that were treated with intravitreal anti-VEGF injections between July 2013 to November 2015. The inclusion criteria were: 1) The patients initially treated with intravitreal Ranibizumab (0.5 mg/0.05 ml) and later switched to Aflibercept (2.0 mg/0.05 ml); 2) fovea involving PED; 3) minimum height of PED of 100 μm (μm) at time of switch, as observed on Spectral Domain OCT (SD-OCT) (Heidelberg Spectralis plus, Heidelberg Germany). Patients were switched to Aflibercept if deemed to have inadequate clinical response (anatomical or functional) to Ranibizumab when assessed clinically. Patients with any other co-existent retinal disease affecting the macula were excluded.

### Treatment protocol

The initial treatment protocol with Ranibizumab consisted of 3 monthly loading doses followed by pro re nata (PRN) schedule. The treatment protocol with Aflibercept consisted of a fixed dosing regimen comprising of monthly injections for first 3 months, followed by 2-monthly injections in first year of treatment. From year two onwards, individualised treatment regime was adopted, either PRN or treat-and-extend (T&E).

### Baseline evaluation

At each visit, the patients were assessed for best corrected visual acuity (BCVA) using the Early Treatment of Diabetic Retinopathy Study (ETDRS) letter score, intraocular pressure, anterior segment assessment and dilated fundoscopy. Patients had fundus fluorescein angiography (FFA) (Topcon 50DX) at baseline and SD-OCT imaging with cube scan at each visit. Selected patients also had indocyanine green angiography (ICG) (Topcon 50DX). FFA was repeated during the follow-up period if deemed necessary.

Baseline characteristics were recorded for age, sex, activity of nAMD and PED reflectivity on OCT. The variables BCVA, PED height, PED width, presence of intraretinal fluid (IRF), subretinal fluid (SRF) and number of injections were measured at baseline (first Ranibizumab) and at 4 other time points: switch (first Aflibercept); 4 months after switch (following the loading phase of 3 monthly Aflibercept); 1 year after switch; and at last follow up visit. The PED height and width were measured manually using the caliper on the OCT software. The PED height was measured as the maximum vertical distance from the base of the RPE to Bruch’s membrane. PED width was measured as the horizontal PED diameter between two points of RPE elevation at the position of greatest PED height. For consistency both measurements were performed on a single SD-OCT cut running through fovea. PED was classified as solid, hollow, or mixed based on reflectivity of material under the RPE on the OCT scans.

### Data collection

The anonymised data were extracted from a single electronic medical records (EMR) system *(Medisoft Ophthalmology, Medisoft Limited, Leeds, UK),* which ensured collection of a standardized dataset throughout the nAMD care pathway. The data entry into EMR was defined prospectively for nAMD treatment which could be extracted retrospectively for audit purposes by authorized users. The standardized data entry and collection ensured its credibility and repeatability than a conventional analysis of the unstructured data in a retrospective chart review. The anonymized database analyses of this type do not require ethical permission as they are viewed as audit or service evaluation (see https://www.hra.nhs.uk/approvals-amendments/what-approvals-do-i-need/). This study was conducted in accordance with the declaration of Helsinki, and the UK’s Data Protection Act.

### Outcome measures

The primary outcomes were assessed for change in BCVA and change in PED height and width from baseline treatment with Ranibizumab to switch to Aflibercept and from switch to all other time points (i.e at 4 months, 12 months and at last follow up). Three paired t-tests were conducted for each of BCVA, PED height and width comparing first Aflibercept to 4 months, 1 year and last follow up visit. In addition, association between the following continuous variables were evaluated using Pearson’s correlations: change in BCVA, PED height and PED width from the time of first Aflibercept to 1 year and BCVA, PED height and PED width at the time of first Aflibercept. Analyses were conducted using SPSS v.24. A significance level of 0.05 was used without adjustment for repeated testing.

## Results

We identified 218 eyes that were switched from Ranibizumab to Aflibercept for the treatment of nAMD during the period July 2013 to November 2015. Fifty eyes met the inclusion criteria. The age, median (range) of these patients was 83 (52–90) years. 16 patients were male and 34 were female. The duration of treatment, mean (SD) [range] with Ranibizumab was 13.2 (4) [3–50] months. The number of intravitreal Ranibizumab injections, mean (SD) [range] received during this time was 7.8 (4.2) [3–21]. The duration of treatment with Aflibercept was 22 (1.2) [6–30] months. The mean number of Aflibercept injections was 11.84 (9.9) [3–19]. The duration of treatment from first Ranibizumab to last follow up was 35.3 (5.2) [12.7–58.3] months (Table 1).

**Table 1:** Baseline characteristics, the mean ± SD [Range] of duration of treatment and number of injections of Ranibizumab and Aflibercept

Age (years) Median (range)	83 (52–90)
Gender (Male: Female)	16:34
Duration of treatment in months	
Treatment with Ranibizumab before switch to Aflibercept	13.2 ± 4 [3–50]
Treatment with Aflibercept after switch	22 ± 1.2 [6–30]
Treatment from first Ranibizumab to last follow up	35.3 ± 5.20 [12.7–58.3]
Number of injections	
Ranibizumab before switch to Aflibercept	7.8 ± 4.2 [3–21]
Aflibercept following switch	11.84 ± 9.9 [3–19]

The PED morphology on SD-OCT at baseline was hollow in 17, solid in 15 and mixed in 18 patients respectively. At the time of switch, the presence of SRF was observed in 32 eyes, IRF in 9 eyes, and both SRF and IRF in 8 eyes along with PED. One eye had PED only with no SRF and IRF.

**Fig. 1:**
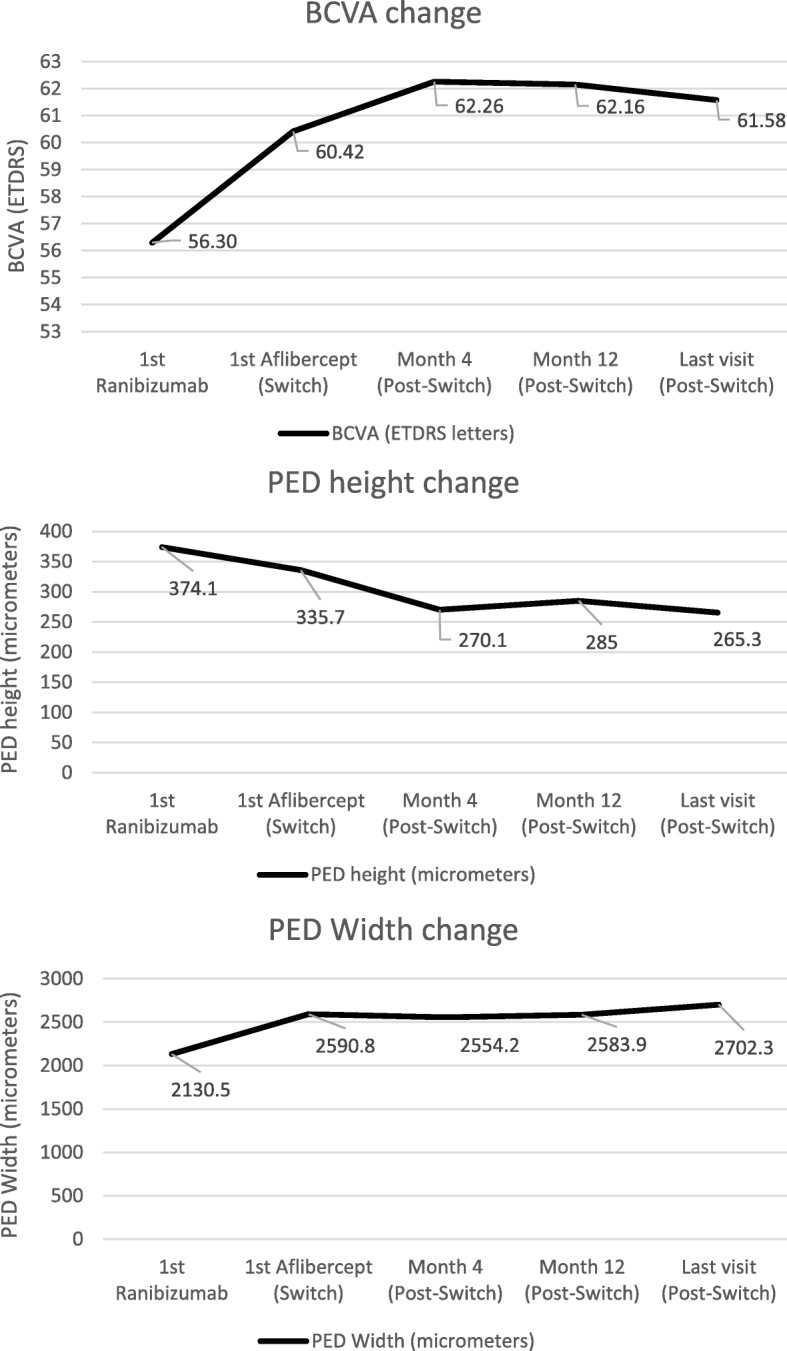
The change in best corrected visual acuity (BCVA), pigment epithelial detachment (PED) height and width mean at five time points

The parameters of BCVA, PED height and PED width mean (SD) at each of the five time points (Table 2). The BCVA improved by 4.12 letters from 56.30 (14.10) to 60.42 (14.90) letters from first Ranibizumab to first Aflibercept. After switch to aflibercept, the BCVA improved by 1.84 (*p* = 0.11), 1.74 (*p* = 0.21) and 1.16 (*p* = 0.45) letters at 4 months, 1 year and the last visit respectively which was not statistically significant at any time point (Fig. 1).

**Table 2:** Descriptive results for BCVA, PED height and PED width as mean (SD) at 1st Ranibizumab, switch to Aflibercept, 4 months, 1 year after switch and at last follow up visit

	Baseline (1st Ranibizumab)	Switch (1st Aflibercept)	4 months after switch	1 year after switch	Last follow-up visit
BCVA	56.30 (14.10)	60.42 (14.90)	62.26 (16.05)	62.16 (15.92)	61.58 (16.40)
PED height	374.1 (166.9)	335.7 (155.4)	270.1 (129.7)	285.0 (146.6)	265.3 (133.3)
PED width	2130.5 (774.0)	2590.8 (819.6)	2554.2 (790.9)	2583.9 (834.8)	2702.3 (847.2)

BCVA in ETDRS letters, PED height and width in micrometers

The PED height reduced by − 38.4μm from 374.1μm (166.9) to 335.7μm (155.4) from first Ranibizumab to the time of switch to Aflibercept. After switch, the reduction in PED height was − 65.6μm (*P* < 0.001), − 50.64μm (*P* = 0.007) and − 68.48μm (P < 0.001) at 4 months, 1 year and at last visit, representing a statistically significant reduction at each time point (Fig. 1).

The PED width increased by + 460.3μm from 2130.5μm (774.0) to 2590.8μm (819.6) on Ranibizumab before switch to Aflibercept. After switch it changed by − 36.6μm (*p* = 0.514), + 29.7μm (*p* = 0.922) and + 118.4μm (*p* = 0.210) at 4 months, 1 year and last visit with no significant difference at any time point (Fig. 1).

Pearson’s correlations were calculated for the change in BCVA, PED height and PED width from the time of switch i.e. first Aflibercept to 1 year and BCVA, PED height and PED width at first Aflibercept (Table 3). Most of the correlations were moderate or low. The change in BCVA at 1 year after switch was not correlated with any of the analyzed anatomical characteristics of PED. There was a moderate negative correlation between reduction in PED height at 1 year after switch and PED height at first Aflibercept (*r* = − 0.474, *p* < 0.05). A moderate negative correlation was identified between PED width and BCVA at the time of switch (*r* = − 0.438, p < 0.05) and a moderate positive correlation was identified between PED width and PED height at switch (*r* = 0.418, p < 0.05).

**Table 3:** Pearson Correlations for BCVA, PED height and PED width at the time of switch i.e. 1st Aflibercept to the change at 1 year from switch

	BCVA change	PED height change	PED width change	BCVA at 1st Aflibercept	PED height at 1st Aflibercept	PED width at 1st Aflibercept
BCVA change	1					
PED height change	−0.181	1				
PED width change	0.095	0.058	1			
BCVA at 1st Aflibercept	−0.216	0.107	0.304*	1		
PED height at 1st Aflibercept	0.114	−0.474**	−0.053	− 0.246	1	
PED width at 1st Aflibercept	0.276	−0.126	−0.271	− 0.438**	0.418**	1

* p < 0.05, ** *p* < 0.01

BCVA measurements in ETDRS letters, PED height and width in micrometers

The change in BCVA, PED height and PED width for lesions of different OCT reflectivity and retinal fluid status was analyzed at the time of first Aflibercept to year one (Table 4). The hollow PED showed most improvement in vision 1 year after switch whereas mixed PED showed most reduction in PED height. The eyes with SRF had most improvement in BCVA and reduction in PED height and increase in PED width compared to eyes with IRF or both IRF and SRF. None of the study eyes had any serious adverse events following anti-VEGF treatment.

**Table 4:** The change in mean (SD) for BCVA, PED height and PED width 1 year after switch to Aflibercept in different PED morphologies and retinal fluid

PED Morphology	Number	BCVA Change (ETDRS letters)	PED height change (micrometers)	PED width change (micrometers)
Hollow	17	3.41 (13.20)	−34.0 (71.1)	−43.6 (414.3)
Mixed	18	0.00 (7.78)	− 91.1 (152.1)	− 0.111 (647.7)
Solid	15	1.93 (6.89)	−20.9 (136.4)	26.6 (385.2)
Retinal Fluid				
None	1	−2.00	6.0	48.0
SRF	32	2.53 (7.28)	− 69.5 (131.5)	93.9 (474.9)
IRF	9	1.78 (7.10)	−2.78 (118.0)	−97.4 (571.8)
SRF and IRF	8	−1.00 (18.67)	−36.3 (118.5)	− 314.9 (419.1)

## Discussion

Our study represents real world anatomical and functional response of PED after switching between two licensed anti-VEGF agents with 1 year or more follow up. Treatment naive AMD lesions are expected to show better functional and anatomic response to anti-VEGF therapy as compared to the switch group due to lack of chronic changes in outer retina. This is reflected with our findings of initial improvement in visual acuity while on treatment with Ranibizumab, which then remained stable after switch to Aflibercept. Although there was a trend to vision improvement at all measured time points after switch, it was not statistically significant. None of the analyzed parameters (BCVA, PED height and width at switch and PED height and width change at 1 year after switch) were found to be correlated with the change in BCVA at 1 year after switch to Aflibercept. We chose the PED height of ≥100 μm in order to be able to appreciate vertical change better on manual measurements. The fact that the PED remains high despite previous Ranibizumab treatment may suggest an active lesion with CNV leakage, or a larger lesion. These higher PEDs showed less decrease in height after switch to Aflibercept representing resistant lesions less likely to respond to further or different anti-VEGF. This may explain the negative correlation found in our study between PED height at time of switch to Aflibercept and change in PED height at 1 year post-switch.

The switch patients in nAMD represent a group which is chronic and refractory to previous treatment. The response to treatment is different than in treatment naïve patients who have new active disease and usually show good outcome with early initiation of anti-VEGF treatment. For treatment naive patients with nAMD related PED, most studies have reported anatomical improvement in terms of reduction in PED height with Bevacizumab [10–13] Ranibizumab [10, 11, 13–22] and Aflibercept [10, 20–22]. With Bevacizumab (1.25 mg) treatment, Freeman WR et al. showed no statistically significant change in SRF and IRF resorption or visual acuity improvement in PED and non-PED groups. [11]. In studies involving ranibizumab, some studies have reported improvement in VA [14, 15] whereas others have reported variable visual results with no correlation between change in BCVA and PED height [16, 17]. Chan et al. demonstrated improvement of visual acuity with higher doses of Ranibizumab (2 mg) at 12 months [18]. A post hoc subgroup analysis in the HARBOR study of patients presenting with PED at baseline reported PED height reduction and BCVA gain with Ranibizumab across PEDs of all heights but a decrease in vision in eyes with extra-large PEDs treated with higher dose of Ranibizumab [19]. With aflibercept treatment H J Cho and Balaskas K have reported greater reduction in PED height compared to ranibizumab [20, 22] at 1 year. They also noticed improved BCVA at 1 year in both groups but variable correlations to baseline parameters. Similar trend of improved anatomical outcomes with aflibercept compared to both ranibizumab and bevacizumab was noticed by A Au at 6 months [10].

Ranibizumab and Bevacizumab have been in use for nAMD for some time, with Aflibercept being introduced later [5, 6]. Switching of treatment to Aflibercept was attempted in patients who were either unresponsive or partly responsive to other drugs. These patients usually had persistent SRF, IRF, PED and some degree of RPE atrophy to retina due to long term anti-VEGF use and disease process itself. Some patients had shown good earlier response but later reached a plateau due to tachyphylaxis or tolerance. Pharmacological difference in drugs and binding abilities to different receptors on retina led way to try Aflibercept in refractory patients. The knowledge and data of its real world effectiveness is currently evolving. The effect of switching treatment from Ranibizumab or Bevacizumab to Aflibercept is currently being reported in recent studies [23–29]. Most studies showed reduction in PED size and reduction in SRF and IRF but the effect of the switch on visual acuity proved to be variable. Some studies reported improvement in vision [23, 24] whereas others reported no change or worsening of vision [25–27]. These studies have assessed smaller height PED [27] or shorter duration of follow up after switch [23, 24]. These observations contribute to early outcomes however it’s difficult to draw parallels and uniform conclusion.

In coherence with other studies, we found decrease in PED height with Ranibizumab and further reduction at month 4 (post loading) with Aflibercept was much more compared to 1 year. The reduction in PED height achieved on Aflibercept was more at all time points than on Ranibizumab before switch. Similar findings were reported by S de Massougnes [27]. Broadhead et al. noticed that spacing of injections from monthly to 2 monthly after loading phase resulted in increase in height, width and length for 1 month after injection compared with 2 months after injection. This implies that such lesions probably need more frequent treatment. We also observed increase in width on Ranibizumab treatment. The width temporarily decreased at month 4 (post loading) after switch but again continued to increase at 1 year and last follow up. This was in contrast to Broadhead et al. who noticed consistent reduction in PED height, width and length at 24 and 48 weeks after switch [28]. The fact that we measured width in the plane of greatest PED height, and not independently of each other, might explain this variability. It could be hypothesised that flattening of lesion broadened the base of lesion due to its mechanical properties. This is more likely to be the case in fibrovascular PED than in serous PED. The BCVA gain in our study was also much less than other studies.

We noticed that improvement of vision and reduction of PED height was maximum in eyes associated with SRF alone. This was probably achieved due to a better control of the CNV activity and leakage. The internal reflectivity of lesion on SD-OCT is believed to be based on content of PED with serous PED being hyporeflective, fibrovascular lesions having mixed reflectivity and solid drusenoid lesions being hyperreflective but a review by Zayit-Soudry, Shiri et al. found this interpretation to be inaccurate [1]. Punjabi et al. found that less reflective PEDs on SD-OCT responded better to anti-VEGF therapies. [30]. In our study, hollow PED showed most improvement in BCVA whereas mixed PED showed most reduction in PED height. These findings were similar to Broadhead et al. [28]. This could be due to drying of serous component in hollow PED and change in morphology of neovascular component in mixed PED. More recently K Balaskas et al. have evaluated change in reflectivity based on pixel intensity of PED content using a novel software [22]. This might be useful in future studies.

The main limitations of our study include its retrospective design and small numbers. The treated PED itself forms a small subset of treated nAMD cases and analyzing switches in treatment in this subgroup generated even fewer yet significant numbers to draw some meaningful interpretation on switching treatment. This information could be useful in designing further studies where power calculations can be done at the start of the study to help decide the length of the study with more number of cases. The assessment of PED parameters was done for height and width only, rather than total internal geometry of lesion. Without accurate data on PED volume it might be hypothesised that reduction in PED height may increase the basal diameter of PED, therefore keeping the PED volume constant. Assessment of PED volume would offer a more accurate picture on the effect of anti-VEGF treatment on PED but the OCT software available to us in clinical practice does not allow for accurate PED volume measurement. Although PED volume can be estimated by mathematical formulation (taking into account PED height and width and assuming the PED has a regular shape), due to the large variability in PED shape in a real world setting such a simplistic calculation would run the risk of inducing a large error in the final results. This prompted us to choose PED height and width together with presence of SRF and IRF as the variables to be included in the final calculations. Some patients may have had progression of cataract and cataract surgery during the treatment however this was not assessed as a part of this study. It is difficult to predict if any visual improvement could be due to cataract surgery. However, no such significant surgical event was recorded in the database.

## Conclusion

Different studies have analysed response to anti- VEGF in PEDs of heights varying from 35 μm to 1400 μm. It is difficult to draw comparisons or pool data from other studies due to different study designs, baseline characteristics and treatment regimes. There is growing evidence that recalcitrant nAMD is associated with outer retinal layer and photoreceptor/RPE damage due to persistent leakage and scarring. Therefore, switching between intravitreal anti-VEGF agents may offer anatomical improvement with further drying and flattening of macula but no functional improvement due to irreversible internal changes in retina. As Aflibercept was licensed after Ranibizumab, we had a substantial number of patients with switch from Ranibizumab to Aflibercept but not vice versa. It would be useful to have a study looking at outcomes after switch from Aflibercept to Ranibizumab for comparison but at present we are limited by numbers and hence could not include it as part of this study. Our paper adds strength to outcomes in switching treatment to different anti-VEGF drug in recalcitrant CNV with PED with substantial follow-up before and after switch. Our study also adds that recalcitrant PEDs which remain high after Ranibizumab treatment may be less likely to respond to further Aflibercept injections.

## Abbreviations

AMD: Age-related macular degeneration
Anti-VEGF: Anti- vascular endothelial growth factor
BCVA: Best Corrected Visual Acuity
EMR: Electronic Medical Records
ETDRS: Early Treatment Diabetic Retinopathy Study
FFA: Fundus Fluorescein Angiography
IRF: Intra-retinal fluid
nAMD: neovascular age-related macular degeneration
OCT: Optical Coherence Tomography
PED: Pigment epithelial detachment
PRN: Pro re nata
SD: Standard Deviation
SD-OCT: Spectral domain optical coherence tomography
SRF: Sub-retinal fluid
T&E: Treat and Extend

## References

[1] Zayit-Soudry, Shiri et al. Retinal pigment epithelial detachment Surv Ophthalmol, 2007;52(3):227–43. 10.1016/j.survophthal.2007.02.008 17472800

[2] Gross-Jendroska M, Flaxel CJ, Schwartz SD (1998). Treatment of pigment epithelial detachments due to age-related macular degeneration with intra-ocular C3F8 injection. Aust N Z J Ophthalmol.

[3] Axer-Seigel R, Ehrlich R, Rosenblatt I (2004). Photodynamic therapy for occult choroidal neovascularization with pigment epithelium detachment in age-related macular degeneration. Arch Ophthalmol.

[4] Nicolo M, Ghiglione D, Lai S, Calabria G (2005). Intravitreal triamcinolone in the treatment of serous pigment epithelial detachment and occult choroidal neovascularization secondary to age-related macular degeneration. Eur J Ophthalmol.

[5] Ranibizumab and pegaptanib for the treatment of age-related - NICE.https://www.nice.org.uk/guidance/ta155.27.Aug.2008.

[6] Aflibercept solution for injection for treating wet age-related macular degeneration - NICE https://www.nice.org.uk/guidance/ta294.

[7] Papadopoulos N, Martin J, Ruan Q (2012). Binding and neutralization of vascular endothelial growth factor (VEGF) and related ligands by VEGF trap, ranibizumab and bevacizumab. Angiogenesis.

[8] Heier JS, Brown DM, Chong V (2012). Intravitreal aflibercept (VEGF trap-eye) in wet age-related macular degeneration. Ophthalmology.

[9] Stewart MW, Rosenfeld PJ (2008). Predicted biological activity of intravitreal VEGF trap. Br J Ophthalmol.

[10] Adrian A, Parikh VS, Singh RP, et al. Comparison of anti-VEGF therapies on fibrovascular pigment epithelial detachments in age-related macular degeneration. Br J Ophthalmol. 2016 Dec 2; 10.1136/bjophthalmol-2016-309434. 10.1136/bjophthalmol-2016-309434 27913442

[11] Freeman WR, Kozak I, Yuson RM (2011). Prognostic implications of pigment epithelial detachment in bevacizumab (avastin)-treated eyes with age-related macular degeneration and choroidal neovascularization. Retina.

[12] Giansanti F, Bacherini D, Giacomelli G et al. Intravitreal anti-VEGF therapy for vascularized pigment epithelium detachment in age-related macular degeneration. Eur J Ophthalmol 2014 May-Jun;24(3):402–408. doi: 10.5301/ejo.5000388. Epub 2013 Nov 12. 10.5301/ejo.5000388 24242217

[13] Baba T, Kitahashi M, Kubota-Taniai M (2012). Two-year course of subfoveal pigment epithelial detachment in eyes with age related macular degeneration and visual acuity better than 20/40. Ophthalmologica.

[14] Chevreaud O, Oubraham H, Cohen SY, Jung C, Blanco-Garavito R, Gherdaoui F, Souied EH (2017). Ranibizumab for vascularized pigment epithelial detachment: 1-year anatomic and functional results. Graefes Arch Clin Exp Ophthalmol.

[15] Arora S, McKibbin M (2011). One-year outcome after intravitreal ranibizumab for large, serous pigment epithelial detachment secondary to age-related macular degeneration. Eye.

[16] Inoue M, Arakawa A, Yamane S, Kadonosono K (2013). Variable response of vascularized pigment epithelial detachments to ranibizumab based on lesion subtypes, including polypoidal choroidal vasculopathy. Retina.

[17] Panos GD, Gatzioufas Z, Petropoulos IK (2013). Effect of ranibizumab on serous and vascular pigment epithelial detachments associated with exudative age-related macular degeneration. Drug Des Devel Ther.

[18] Chan CK, Abraham P, Sarraf D (2015). Earlier therapeutic effects associated with high dose (2.0 mg) Ranibizumab for treatment of vascularized pigment epithelial detachments in age-related macular degeneration. Eye (Lond).

[19] Sarraf D, London NJ, Khurana RN (2016). Ranibizumab treatment for pigment epithelial detachment secondary to Neovascular age-related macular degeneration: post hoc analysis of the HARBOR study. Ophthalmology.

[20] Cho HJ, Kim KM, Kim HS (2016). Response of pigment epithelial detachment to anti-vascular endothelial growth factor treatment in age-related macular degeneration. Am J Ophthalmol.

[21] Dirani A, Ambresin A, Marchionno L et al. Factors influencing the treatment response of pigment epithelium detachment in age-related macular degeneration. Am J Ophthalmol 2015 Oct;160(4):732–8.e2. doi: 10.1016/j.ajo.2015.06.025. Epub 2015 Jul 2. 10.1016/j.ajo.2015.06.025 26144701

[22] Balaskas K, Karampelas M, Horani M (2017). Quantitative analysis of pigment epithelial detachment response to different anti-vascular endothelial growth factor agents in wet age-related macular degeneration. Retina.

[23] Kumar N, Marsiglia M, Mrejen S (2013). Visual and anatomical outcomes of intravitreal aflibercept in eyes with persistent subfoveal fluid despite previous treatments with ranibizumab in patients with neovascular age-related macular degeneration. Retina.

[24] Patel KH, Chow CC, Rathod R (2013). Rapid response of retinal pigment epithelial detachments to intravitreal aflibercept in neovascular age-related macular degeneration refractory to bevacizumab and ranibizumab. Eye (Lond).

[25] Gharbiya M, Iannetti L, Parisi F (2014). Visual and anatomical outcomes of intravitreal aflibercept for treatment-resistant neovascular age-related macular degeneration. Biomed Res Int.

[26] Grewal DS, Gill MK, Sarezky D (2014). Visual and anatomical outcomes following intravitreal aflibercept in eyes with recalcitrant neovascular age-related macular degeneration: 12-month results. Eye (Lond).

[27] He L, Silva RA, Moshfeghi DM, Blumenkranz MS, Leng T (2016). Aflibercept for the treatment of retinal pigment epithelial detachments. Retina.

[28] Broadhead GK, Hong T, Zhu M, Li H, Schlub TE, Wijeyakumar W, Chang AA (2015). Response of pigment epithelial detachments to intravitreal aflibercept among patients with treatment-resistant neovascular age-related macular degeneration. Retina.

[29] de Massougnes S, Dirani A, Ambresin A (2016). Pigment epithelial detachment response to aflibercept in neovascular age-related macular degeneration refractory to ranibizumab: time course and drug effects. Retina.

[30] Punjabi OS, Huang J, Rodriguez L (2013). Imaging characteristics of neovascular pigment epithelial detachments and their response to anti-vascular endothelial growth factor therapy. Br J Ophthalmol.

